# Development of Ternary and Quaternary Catalysts for the Electrooxidation of Glycerol

**DOI:** 10.1100/2012/502083

**Published:** 2012-05-01

**Authors:** L. M. Artem, D. M. Santos, A. R. De Andrade, K. B. Kokoh, J. Ribeiro

**Affiliations:** ^1^Department of Chemistry, Federal University of Espírito Santo, 29075-910 Vitória, ES, Brazil; ^2^Department of Chemistry, São Paulo University, 14040-901 Ribeirão Preto, SP, Brazil; ^3^Equipe Electrocatalyse, LACCO, UMR 6503 Université de Poitiers, 4 rue Michel Brunet, B27-BP 633, 86022 Poitiers Cedex, France

## Abstract

This work consisted in the preparation of platinum-based catalysts supported on carbon (Vulcan XC-72) and investigation of their physicochemical and electrochemical properties. Catalysts of the C/Pt-Ni-Sn-Me (Me = Ru or Ir) type were prepared by the Pechini method at temperature of 350°C. Four different compositions were homemade: C/Pt_60_Sn_10_Ni_30_, C/Pt_60_Sn_10_Ni_20_Ru_10_, C/Pt_60_Sn_10_Ni_10_Ru_20_, and C/Pt_60_Sn_10_Ni_10_Ir_20_. These catalysts were electrochemically and physically characterized by cyclic voltammetry (CV), chronoamperometry (CA) in the presence of glycerol 1.0 mol dm^−3^, X-ray diffraction (XRD), and high-resolution transmission electron microscopy (HRTEM). XRD results showed the main peaks of face-centered cubic Pt. The particle sizes obtained from XRD and HRTEM experiments were close to values ranging from 3 to 8.5 nm. The CV results indicate behavior typical of Pt-based catalysts in acid medium. The CV and CA data reveal that quaternary catalysts present the highest current density for the electrooxidation of glycerol.

## 1. Introduction

Biofuels have become important worldwide due to the global demand for clean and renewable energy. Fuel cells are electrochemical devices in which reagent insertion converts energy directly into electrical energy and heat via the chemical energy of the fuel half-reactions. As a result, their efficiency is much higher as compared to traditional methods of power generation, since it is nonmechanical and has no thermodynamic limitations [1]. Unlike batteries, which are an energy storage devices limited by the amount of reagent that is available, the fuel cell converts the energy generated from fuel oxidation into an orderly flow of electrons.

From a theoretical viewpoint, as long as the fuel is supplied fuel cell is capable of producing energy [1–6]. However, the operational lifetime of fuel cells is reduced due to factors such as electrocatalytic activity (poisoning) and proton conductivity of the electrodes, comprising them [2, 5]. Therefore, anodes must have increasingly better catalytic activity and corrosion resistance, in order to improve cell efficiency.

There is a great interest in cells that use ethanol as fuel. Indeed, Brazil is one of the largest world producers of ethanol, which has much lower toxicity as compared to methanol and for which technology very similar to that of the methanol cells can be employed [1, 5].

Due to the difficulty in breaking the C–C bond of ethanol at low temperatures, the main products of its electrooxidation reaction are acetaldehyde and acetic acid or acetate, which leads to low faradaic efficiency (17–33% of the theoretical energy) and production of compounds with no practical value [7]. The use of polyols as fuel can be a sustainable alternative. Polyols such as ethylene glycol and glycerol are less toxic and volatile than methanol and have a relatively high theoretical energy density, 5.2 and 5.0 kWh/kg, respectively, against energy densities of 6.1 and 8.0 kWh/kg for methanol and ethanol, respectively [7]. In addition, each of these carbon compounds carrys a group of alcohol whose partial oxidation to oxalate and mesoxalate without cleavage of the C–C bond for production of carbonate culminates in a flow of 8 and 10 mol of electrons per mol of ethylene glycol and glycerol, respectively, as compared to 6 and 12 mol of electrons for methanol and ethanol, respectively, for complete oxidation [8–11]. Thus, the possibility of oxidizing alcohol groups without breaking the C–C bond may result in 70 to 80% of the total energy available for ethylene glycol and glycerol. However, only glycerol can be obtained from biomass, and ethylene glycol is mainly produced by oxidation of ethylene. Glycerol is mainly generated from the methanolysis of vegetable oils so indirectly it is a natural byproduct. The growing demand for methyl esters as fuel additives has promoted increased production of glycerol, which has thus become a relatively cheap raw material [7, 10, 11].

Platinum catalysts are already known for being the best for the dissociative adsorption of small organic molecules, including ethanol and glycerol. However, its catalytic activity is limited to the cleavage of the C–C bond, since only two-carbon intermediates have been identified in the oxidation of ethanol with platinum catalysts. In a search for better selectivity for the production of CO_2_ and higher catalytic activity, several metallic alloys have been investigated [1, 6, 11, 12].

This work aims to produce platinum-based catalysts and study their physicochemical and electrochemical characteristics, in order to enhance the catalytic efficiency. With a view to reducing the cost of catalysts, the inclusion of lower-cost metals such as ruthenium, tin-and nickel as compared to platinum is also analyzed.

## 2. Experimental Section

Catalysts of the C/Pt-Ni-Sn-Me (Me = Ru or Ir) type were prepared by the Pechini method [13]. This method is based on the synthesis of resins from metal precursors (metal chloride) and citric acid (Merck) in a mixture with ethylene glycol (Merck), at a molar ratio of 1 : 4: 16, respectively. After the dissolution of citric acid in ethylene glycol (at 60–65°C), and metal chloride (H_2_PtCl_6_, IrCl_3_·*x*H_2_O, RuCl_3_·*x*H_2_O, NiCl_2_; Sigma-Aldrich) dissolved in isopropanol (Merck) was added, and the temperature was increased to 85–90°C for the esterification step. Finally, the mixture was kept under vigorous stirring for 1–2 h. The metal concentration for each resin was determined using inductively coupled plasma (ICP-OES model optima 7300 V).

The homemade catalysts consisting of 40 wt. % metal and 60 wt. % carbon (Vulcan XC-72) were prepared by mixing the appropriate amounts of each metal resin together. Then, the catalysts were dispersed in 2 mL ethanol (Sigma-Aldrich) for 20 minutes, in ultrasonic bath (Thornton model T14). Next, the solvent was evaporated in an oven at 80°C, which was followed by annealing at 350°C for 3 hours (Microprocessor Controlled Oven model Q318M/QUIMIS). Four catalysts were prepared, with the following compositions: C/Pt_60_Sn_10_Ni_30_, C/Pt_60_Sn_10_Ni_20_Ru_10_, C/Pt_60_Sn_10_Ni_10_Ru_20_, and C/Pt_60_Sn_10_Ni_10_Ir_20_.

The physicochemical characterization of the material was carried out by high resolution transmission electronic microscopy (HRTEM) on a JEOL/JEM-3010 microscope operating at 300 kV. TGA and DTA analyses using a TA instrument model SDT Q600 V20.9 build 2.0, in dry air, at a heating rate of 5°C/minute from 25 to 550°C was accomplished. X-Ray diffraction (XRD) analysis was performed on a Shimadzu XRD model 6000 instrument using CuK*α* radiation (*λ* = 1.5406 Angstrom). The following parameters were kept constant during all the tests: 2*θ* = 20° to 90°, step = 0.03°, and duration of each analysis of 1.97 h. The particle size, *d*, was calculated by means of the Scherrer equation [14], where *λ* is the radiation wavelength, B is the reflection width at half-maximum intensity (FWHM), and *θ* is the angle at the maximum intensity:

(1) $$\begin{array}{c} d=\frac{0.9\;\times \;\lambda }{B\;\times \;\cos\theta }. \end{array}$$

All the solutions employed in this work were prepared with 18 MΩ cm water produced and purified in a Reverse Osmosis System (Puritech model RO 300). Electrochemical studies were conducted by using 0.5 mol·dm^−3^H_2_SO_4_ (Sigma-Aldrich) as the supporting electrolyte. The electrochemical experiments were carried out in a one-compartment cell with a main body of 30 mL. The working electrode was prepared by starting from a black ink consisting of 1 mg of the catalyst, 5 *μ*L Nafion (Sigma-Aldrich), and 95 *μ*L ethanol, then; the solution was placed in ultrasonic bath for a period of 20 minutes, followed by deposition from a micropipette onto a freshly polished glassy carbon substrate and the drying in oven at 60°C for 20 minutes. The carbon electrode was utilized as counterelectrode, and an Ag/AgCl_sat⁡_  reference electrode was employed and positioned close to the working electrode. The electrochemical activity of the catalysts was assessed by chronoamperometry in the presence of glycerol (Sigma-Aldrich) 1.0 mol·dm^−3^ at 400 mV versus Ag/AgCl_sat⁡_ for 2 hours. The electrochemical experiments were performed using an AUTOLAB potentiostat/galvanostat model 302N.

## 3. Results and Discussion

**Figure 1:**
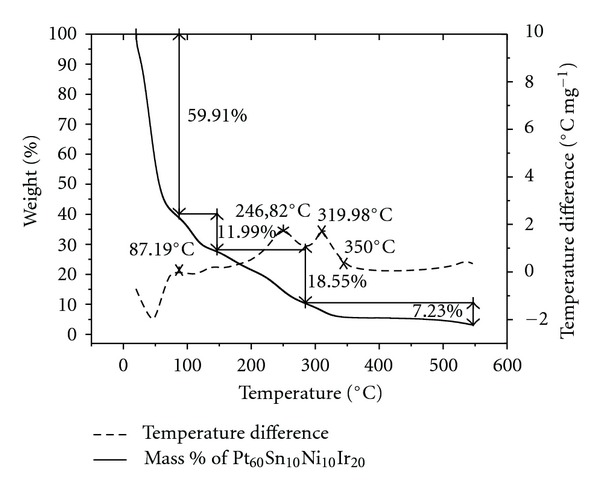
TGA curves obtained for the C/Pt_60_Sn_10_Ni_10_Ir_20_ catalysts at a heating rate of 5°C min^−1^ from room temperature to 550°C.

The representative TGA and DTA curves obtained for the C/Pt-Sn-Ni-Me catalysts are presented in Figure 1. This technique monitors the mass loss of the catalyst as a function of the temperature. The results show that there is mass reduction beginning at ca. 87°C, with total mass loss of ~60% for all the investigated catalysts. Moreover, there are two other mass loss processes: one localized at ca. ~245°C and the second located at ca. ~320°C, which continues until ca. 350°C. Thereafter, the mass remains practically constant. These processes are associated with the thermal decomposition of the polymer formation. The TGA study helped the monitoring of three parameters of the catalyst. Firstly, it aided in the selection of the calcinations temperature range (350 to 450°C), in which there was no change in the mass of the catalyst. Secondly, it indicated the temperature 350°C at which all the organic compounds had been removed. Finally, it helps establish the lower temperature at which the smallest particle size is ensured, in order to increase the surface area and consequently promote greater catalytic efficiency.

**Figure 2:**
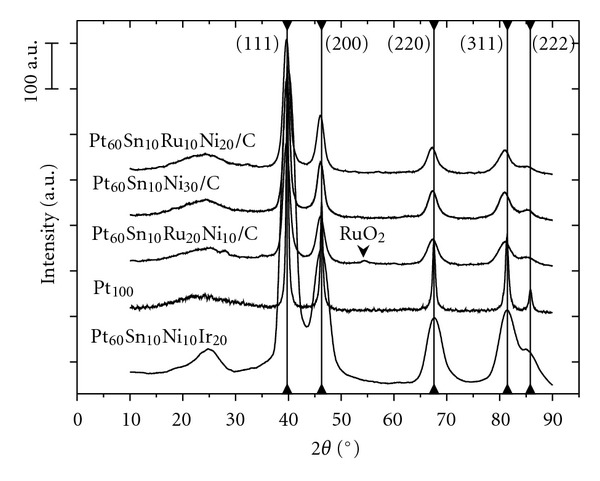
XRD patterns obtained for the different Pt-based catalysts (40% wt. metal loading on carbon) prepared at 350°C for 3 hours.

Figure 2 shows the XRD patterns obtained for the different Pt-based electrocatalysts prepared at 350°C for 3 hours. All the catalysts display a smooth peak at *θ* = 24.3°, attributed to carbon with the reflection plane (002). The other peaks are characteristic of the structure of the face-centered cubic (fcc) structure of metallic platinum and refer to the reflections planes (111), (200), (220), (311), and (222) [15, 16]. Moreover, the formation of ruthenium oxide to a small extent can be noticed from the angle *θ* = 54.6° for Pt_60_Sn_10_Ni_10_Ru_20 _ catalyst.

**Figure 3:**
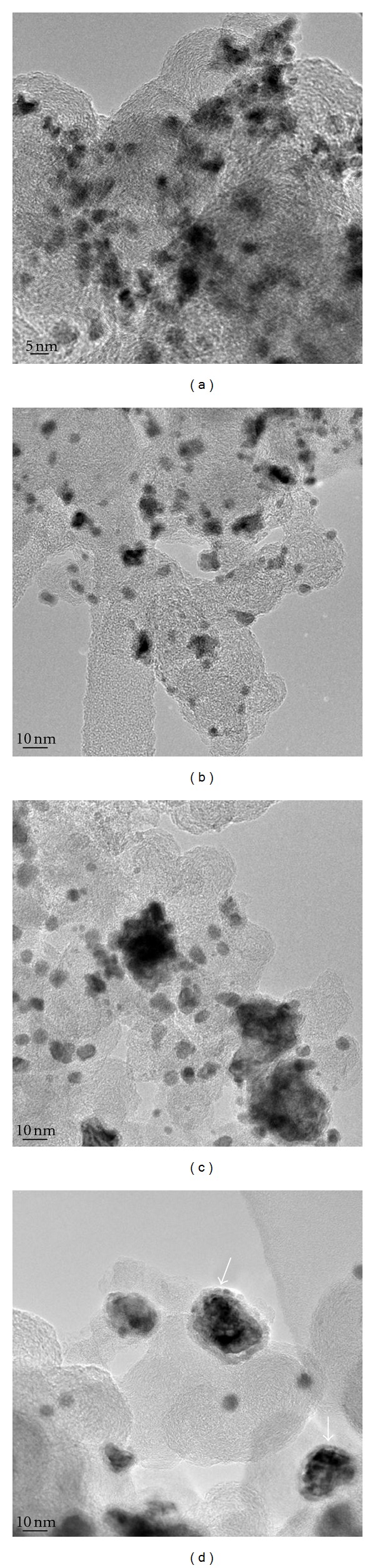
HRTEM images of the Pt-based electrocatalysts: (a) C/Pt_60_Sn_10_Ni_10_Ru_20_, (b) C/Pt_60_Sn_10_Ni_20_Ru_10_, (c) C/Pt_60_Sn_10_Ni_30 _and (d) details of the ring observed on C/Pt_60_Sn_10_Ni_30_ catalyst.

The HRTEM images are depicted in Figure 3. The HRTEM analysis recalls the distribution of the metals on the carbon support. It is expected that the catalyst with homogeneous distribution of the metallic phase also has increased catalytic activity. For the C/Pt_60_Sn_10_Ni_10_Ru_20_ catalyst, the results show that the metallic phase is in the form of spherical particles with sizes ranging from 3 to 10 nm. The particles are distributed throughout the carbon support in a heterogeneous way. In the case of the C/Pt_60_Sn_10_Ni_20_Ru_10_ catalyst, the particles sizes lie between 4 and 7 nm. It can be seen that some are large while others are more scattered than those presented in Figure 3(a). The energy dispersive X-ray analysis (EDX) of the aggregates and individual particles shows the presence of the four metals almost all the time, and their proportions are more homogenous than in the C/Pt_60_Sn_10_Ni_10_Ru_20_ catalyst.

Concerning the C/Pt_60_Sn_10_Ni_30_ catalyst, the metallic phase is present in three different ways: small particles of about 4–6 nm distributed throughout the support (see Figure 3(c)), large particles measuring from 10 to 20 nm and surrounded by small, scattered particles of about 4 nm on average (Figure 3(d)). The EDX analysis of the metallic phase demonstrates the presence of three metals. The proportion of aggregates and large particles is homogeneous, and the order is 60 : 10 : 30 Pt/Sn/Ni. However, small particles are rich in nickel and poor in tin. Finally, the ring observed in Figure 3(d) is rich in nickel. Based on CA experiments done in the C/Pt_60_Sn_10_Ni_30_ electrocatalyst one can infer that the instability and low activity for the glycerol oxidation observed for this electrocatalyst suggests that the Ni segregation can be associated, but this is a hypothesis and further investigations should be made to draw any conclusion.

Table 1 lists the particle size results obtained by XRD and the average particle size obtained by HRTEM for the different catalysts investigated here. The particle size values range from 3.0 to 8.5 nm and are corroborated to each other by both XRD and HRTEM. The catalytic activity is explained by the fact that the activity of a good composition is closely related to the formation of alloys and/or the homogeneous distribution of the components.

**Figure 4:**
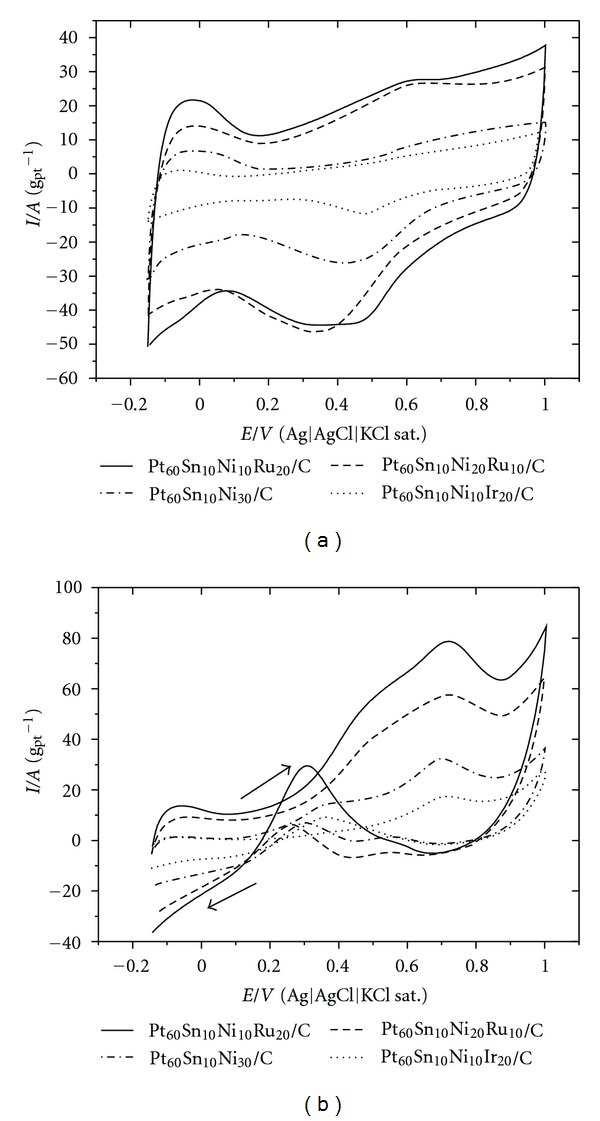
(a) Cyclic voltammograms for the 40 wt.% C/Pt-Sn-Ni-Me catalysts (after 50 cycles) in 0.5 mol.dm^−3^ H_2_SO_4_ solution and (b) in the presence of 1.0 mol dm^−3^ glycerol.

Figure 4(a) illustrates the voltammetric curves obtained for the freshly prepared C/Pt-Sn-Ni-Me catalysts. The voltammetric curves are characterized by the presence of a region related to the desorption/adsorption of hydrogen onto Pt sites. This region is distorted as compared to pure Pt, and this is associated with the presence of transition metals such as ruthenium, and iridium, osmium [17]. Moreover, the presence of transition metals leads to a larger double-layer region in these catalysts, which has been observed before [15, 18].

Figure 4(b) displays the cyclic voltammograms obtained for the C/Pt-Sn-Ni-Me catalysts in the presence of 1.0 mol·dm^−3^ glycerol. The onset of the glycerol oxidation current at quaternary catalysts takes place at ~0.20 V versus Ag/AgCl_sat⁡_. The quaternary catalysts (e.g., C/Pt_60_Sn_10_Ni_10_Ru_20_) containing Ru perform better than quaternary catalysts containing Ir in terms of glycerol oxidation. For all the catalysts investigated in this work, there are at least four oxidation peaks: two in the positive-going scan and two in the negative-going scan. These peaks are related to the different organic species adsorbed onto the platinum-based nanoparticles, culminating in the formation of distinct intermediates, as discussed in the literature [6, 14].

**Figure 5:**
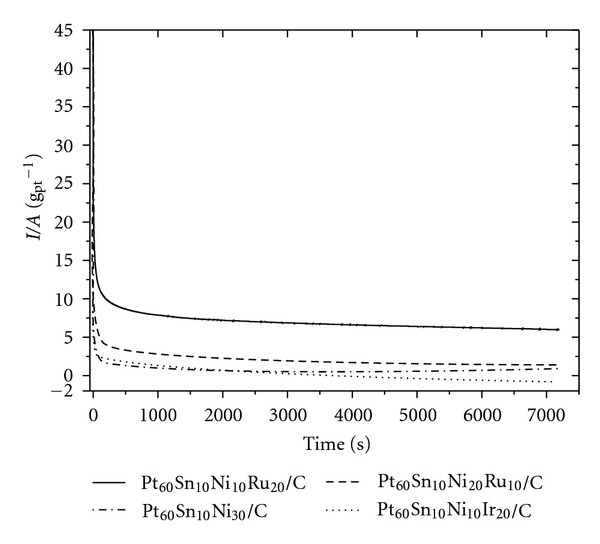
Current versus time curves for the glycerol oxidation catalysts in H_2_SO_4_ supporting electrolyte 0.5 mol·dm^−3^ in the presence of 1.0 mol·dm^−3^ glycerol.

Figure 5 represents the chronoamperometry (CA) curves recorded at a constant potential of 0.4 V versus Ag/AgCl_sat⁡_ for two hours. CA allowed evaluation of the electrocatalytic activity of the catalysts. The C/Pt_60_Sn_10_Ni_10_Ru_20 _ catalyst furnished the best result in this analysis.

Finally, the calculation of the cost/benefit ratio was carried out for the catalysts, by considering only the value of the metallic precursor, since the other reagents were used in equal amounts in all catalysts. The results are summarized in Table 2. The value represented in the second column refers to the cost of obtaining one gram of the metal from its metallic precursor, which was calculated by considering how much metal could be achieved from the precursor. Then, the normalization by gram of each metal was accomplished.

The cost of the catalysts was calculated by considering that all have mass of 1 g of platinum. In the last column, the cost/benefit ratio was computed by dividing the cost of each catalyst by the value of current measured at one hour of CA analysis. This ration was also normalized by one gram of platinum.

The data attest that the best catalyst is the quaternary C/Pt_60_Sn_10_Ni_10_Ru_20_ although it is not the catalyst with the lowest cost. Nevertheless, it affords the best cost/benefit ratio and therefore is the best catalyst in this work.

## 4. Conclusions

The C/Pt-Ni-Sn-Me (Me = Ru or Ir) catalysts were prepared by the Pechini method, and physicochemical characterization and electrochemical properties have been studied by HRTEM, EDX, XRD, and electrochemical techniques. The HRTEM images showed that the particle sizes of catalysts were ranging from 3 to 8.5 nm. EDX analyses showed a good relationship between the nominal and the experimental composition of the C/Pt-Ni-Sn-Me (Me = Ru or Ir) catalysts.

The onset potential for the glycerol oxidation was found to be ~0.20 V versus Ag/AgCl_sat⁡_ for the quaternary catalysts. The current values observed for those catalysts indicate that activation takes place at the catalysts surface, which can be associated with the added Ni metal. The Pechini method proved to be an efficient route for the preparation of catalysts. Furthermore, the development of quaternary catalysts is promising because they have higher efficiency as compared to ternary catalysts, as well as better cost/benefit.

## Figures and Tables

**Table 1:** Particle size obtained from XRD and HRTEM analyses.

Composition of the catalyst (mol %)	*d* (nm)				
	(111)	(200)	(220)	(311)	HRTEM (nm)
Pt_60_Ni_30_Sn_10_	8.5	7.4	5.6	6.1	4–6
Pt_60_Ni_10_Sn_10_Ru_20_	7.2	6.1	4.8	4.2	3–4
Pt_60_Ni_20_Sn_10_Ru_10_	7.6	6.8	4.9	4.9	4–7
Pt_60_Ni_10_Sn_10_Ir_20_	5.3	4.7	4.4	4.1	—

**Table 2:** Cost/benefit ratio of the catalysts.

Metallic precursor	Cost of the metal from precursor (US $/g)	Composition of the catalyst (mol %)	I/A (g_Pt_^−1^) on t = 1 h	Cost of the catalyst (US $/g)	Cost/benefit US $/A
PtCl_4, _Sigma-Aldrich	728.85				
IrCl_3_·nH_2_O, Sigma-Aldrich	980.75	Pt_60_Sn_10_Ni_30_	0.69	1222.83	1772.22
RuCl_3_·nH_2_O, Acros Organics	74.75	Pt_60_Sn_10_Ni_10_Ru_20_	6.71	1250.66	186.39
NiCl_2_ 98%, Sigma-Aldrich	5.64	Pt_60_Sn_10_Ni_20_Ru_10_	1.96	1236.82	631.03
SnCl_4_ 99%, Sigma-Aldrich	1.69	Pt_60_Sn_10_Ni_10_Ir_20_	0.10	1635.34	16353.40
